# Biomarking and Induction of Apoptosis in Ovarian Cancer Using Bifunctional Polyethyleneimine-Caged Platinum Nanoclusters

**DOI:** 10.3389/fonc.2022.898917

**Published:** 2022-06-03

**Authors:** Mengjun Zhang, Haodi Yue, Yuan Liu, Hao Li, Yue Yin, Zhenxing Sun, Ping Cui, Fei Li, Xiuwei Chen, Xin Huang

**Affiliations:** ^1^ Department of Gynecology, Harbin Medical University Cancer Hospital, Harbin, China; ^2^ Department of Center for Clinical Single Cell Biomedicine, Henan Provincial People’s Hospital, Zhengzhou, China; ^3^ Department of Light Chemical Engineering, School of Textiles, Zhongyuan University of Technology, Zhengzhou, China

**Keywords:** ovarian cancer, PEI-Pt NCs, apoptosis, bioimaging, nanomedicine

## Abstract

According to the 2020 GLOBOCAN Global Cancer Women’s Cancer Data, ovarian cancer is the eighth most common tumor in humans. Still, its mortality rate ranks first among all gynecological tumors, with a 5-year survival rate of 30% to 50%. Widespread clinical use of platinum-based drugs has improved survival outcomes in patients with ovarian cancer, but organ toxicity and drug resistance hinder their anticancer effects. In particular, the resistance to platinum drugs is an important reason for ovarian cancer’s high recurrence rate and mortality. With the development of chemotherapeutic drugs synthesized by nanomaterials in the biomedical field, we developed bifunctional ultrafine polyethyleneimine caged platinum nanoclusters (PEI-Pt NCs) to improve the dilemma of platinum drugs. This study aimed to elucidate the antitumor effect of PEI-Pt NCs in OC. First, as observed by confocal microscopy, Pt NCs entered OC cells in a dose-dependent manner and accumulated on the surface of the nuclear membrane and in the nucleus. Subsequently, through cck8, ki-67 immunofluorescence, wound healing assay, transwell assay, clone formation assay, flow cytometry, tunel staining, and western blotting assay, it was confirmed that PEI-Pt NCs could inhibit the proliferation and migration and induce the apoptosis of ovarian cancer cells. PEI-Pt NCs can be used as fluorescent markers for systemic bioimaging of ovarian cancer, showing great potential in diagnosing and treating ovarian cancer, and making a specific contribution to solving the dilemma of platinum-based drug therapy for OC.

## Introduction

Over the past 20 years, ovarian cancer, which accounts for 3% of women’s cancers, has declined by less than 1%, and mortality rates have remained unchanged (1). Along with lung, breast, colorectal, and pancreatic cancers, ovarian cancer is one of the top five causes of cancer death in women (2). When ovarian cancer occurs, surgical tumor cell reduction to R0 is the mainstay of treatment, followed by a combination of platinum-based chemotherapy (3). However, platinum-resistant relapse influences poor prognosis, with 70% of these patients relapsing within 3 years of initial treatment (4). As one of the most representative platinum-based chemotherapeutic agents, cisplatin (II-valent platinum) has many disadvantages, including high plasma clearance, poor specificity, multi-organ toxicity, and myelosuppression (5). Advances in nanotechnology have provided new ideas for developing novel drugs and delivery systems to improve the defects of platinum-based drugs, including the development of platinum-based nanoparticles and non-precious metal platinum-based nanoparticles. Rapid advances in nanomedicines will likely change the paradigm of platinum-based drug therapy for ovarian cancer, significantly improving the effects of such platinum-based drugs and improving survival rates in what is universally considered fatal cancer (6–8).

Research areas related to nanotechnology are alarming, and nanomedicine uses nanomaterials and nanotechnology to design new drugs and delivery systems to improve the efficacy of existing therapies (9–12). In cancer research, nanomedicines have the advantage of tiny size and excellent drug release profiles that give them great potential to overcome the shortcomings of current chemotherapeutic drugs. Most importantly, nanomaterials can improve the efficacy of chemotherapeutic drug-loaded medicines and reduce the toxic side effects of the medications through specific targeting. In addition, the convergence of nanotechnology and medicine has led medical researchers to develop more advanced and innovative nanomedicines that have the potential to achieve versatile diagnostics and therapeutics in parallel. The simultaneous combination of diagnosis and treatment of cancer has become a new strategy for the safe and effective management of cancer (13). Combines targeted drug delivery with diagnostic imaging to achieve improved diagnosis while achieving tumor-specific drug delivery and reducing lethal effects on normal tissue (14, 15). Develop nanomedicine carriers such as liposomes, polymeric nanoparticles, dendrimers, and nano-micelles to effectively diagnose and treat cancer (16–18). Cancer nano drugs offer new opportunities as an innovative and promising new strategy for early detection, improved treatment, and accurate disease diagnosis. It is believed that the combination of nanotechnology and medicine will change the paradigm of cancer management shortly, with cross-collaboration and multidisciplinary convergence being the best option to solve various dilemmas in cancer treatment.

Polyethyleneimine (PEI)-based nanocarriers are promising drug delivery systems with the advantages of efficient gene transfection, negligible toxicity, ability to deliver nucleic acids and chemotherapeutic drugs, easy modification with targeted molecules, and good responsiveness to external stimuli. However, its application in drug delivery systems is still lacking, and it is mainly applied to the construction of gene vectors (19–21). During the study of drug delivery based on polymer assemblies, PEI was found to promote the selective accumulation of platinum into cancer cells while reducing toxic side effects (22). Until to now, PEI has been progressed in preclinical studies. For example, PEI can be used as a ligand for N-heterocyclic carbine platinum to form NHC-Pt-PEI conjugates to induce human cancer cell death *in vitro* and *in vivo (* 23). NHC-Pt-PEI conjugates were shown to inhibit the growth of HCT116 xenograft tumors without significant side effects compared to the widely used oxaliplatin (23). It has also been demonstrated that UCNP@PEI-Phen-Pt-PEG-RGD nanocomposites have cytotoxic effects on human laryngeal cancer cell lines and may integrate imaging and cancer therapy as one (24). Researchers have also confirmed that the polymeric polyethyleneimine drug delivery system can inhibit the growth of SKOV3 cells. Thus it is a highly desirable drug delivery system that can be used to develop novel drugs dedicated to cancer therapy (19).

In this study, fluorescent ultrafine polyethyleneimine (PEI) caged platinum nanoclusters (PEI-Pt NCs, Pt NCs) were synthesized by a simple one-step reduction method, which was used as fluorescent carriers for bioimaging of ovarian cancer system cells and selectively inhibited malignant biological behaviors such as proliferation, migration and clone formation of ovarian cancer cells. Meanwhile, an effective anti-cancer drug for ovarian cancer can specifically induce apoptosis of ovarian cancer cells. Our PEI-Pt NCs have a dual function to enable the treatment of ovarian malignancies as an anti-cancer chemotherapeutic agent accompanied by fluorescence imaging of cancer cells during drug release.

## Materials and Methods

### Cell Culture

Ovarian cancer cell lines were purchased from the Cell Resource Center of Shanghai Institutes of Biological Sciences (Shanghai, People’s Republic of China). Cells were cultured at 37°C; 5% CO2 in a mixed 1640 cell culture medium (10% fetal bovine serum, 100 U/mL penicillin, and 100 ug/mL streptomycin). PEI-Pt NCs at 0.05ug/mL and 0.1ug/mL were added to the cells respectively and continued to be cultured for 24h before collecting the cells for subsequent experiments. The number of cell passages was kept constant during the investigation and the experiment was repeated three times under the same conditions.

### Detection of the Uptake of PEI-Pt NCs

Synthetic procedure of PEI-Pt NCs are detailed in **Supplementary Materials**. IOSE80, A2780 and SKOV3 cells were cultured in glass culture dishes for 24 h. PEI-Pt NCs (0.1 ug/mL) were incubated for 4 h, and then their ability to enter the cells was determined. 4% paraformaldehyde was fixed and washed three times with PBS, and then the nuclei were stained for 10 min at room temperature (4’,6-diamidino-2-phenylindole, DAPI, Solarbio, Beijing, China). PBS was washed three times. Fluorescence imaging was performed with a confocal microscope (LSM830, Zeiss, GER) at 488/560 nm (excitation/emission).

### CCK-8 Assay

The proliferation inhibition of PEI-Pt NCs, cisplatin (Qilu Pharmaceutical, China) in IOSE80, A2780 and SKOV3 cells. IOSE80, A2780 and SKOV3were incubated in 96-well plate culture dishes for 24 h and then incubated with different concentrations of PEI-Pt NCs and cisplatin for another 24 h. Finally, CCK-8 (Abmole Bioscience, USA) was added for 2 h, and then the optical density of each well was calculated at 450 nm (OD, EN sight, PerkinElmer, USA). The cells’ semi-inhibitory concentration (IC_50_) was calculated using the CCK-8 colorimetric method and statistical analysis was performed using GraphPad Prism 9.0.

### Western Bolt Assay

Western blot was used to detect the protein expression levels of apoptosis-related genes (BAX, BCL-2, P53 and Caspase-3) in three groups of 0 ug/mL, 0.05 ug/mL and 0.1 ug/mL. Antibodies were purchased from CST (Cell Signaling Technology), total intracellular proteins were collected by radioimmunoprecipitation analysis of lysis buffer, and an enhanced BCA protein analysis kit detected protein concentrations. SDS-gel preparation, sample loading, electrophoresis, and membrane transfer. Subsequently, PVDF membranes were incubated with primary antibodies overnight, washed and incubated with secondary antibodies for 1h, and finally detected with the ECL (Yakinase) chemiluminescence kit.

### Flow Cytometry for Cell Apoptosis Analysis

A2780 and SKOV3 cells were cultured at a density of 1000/mL per group in 6-well culture dishes for 24 h. Pt NCS at 0.05 ug/mL and 0.1 ug/mL were added for 4 h. Cells were collected by centrifuging tubes, washed three times with PBS, and then stained with Annexin V/PI. Fluorescence emission was detected by flow cytometry at excitation wavelengths of 530 nm and 575 nm. Data were processed with Flow JO 10.5.

### Ki67 Immunofluorescence

Cells were fixed in 4% paraformaldehyde for 5 min at 4°C, washed 3 times with phosphate, and then closed with 5% skim milk and 0.1% Triton X-100 at room temperature for 1 h. Climbing slices were incubated with anti-Ki67 primary antibody (1:200; Sigma-Aldrich; Merck KGaA) at 4°C overnight and then incubated with Alexa Fluor-labeled secondary antibody (1:500; Sigma-Aldrich; Merck KGaA) for 1 h and washed with PBS. The nuclei were stained with DAPI at a constant temperature of 4°C for 10 min. Staining was observed with an immunofluorescence microscope (BX60; Olympus Corporation, Tokyo, Japan) at three random fields of view.

### Transwell Assay

A2780 and SKOV3 cells were inoculated with 1×10^5^ cells/well in 200 uL of serum-free culture medium, and Pt NCs (0 ug/mL, 0.05 ug/mL and 0.1 ug/mL) were added to the upper chamber of the transwell (Corning, USA). Meanwhile, 700 uL of medium containing 15% fetal bovine serum was added to the lower chamber of the 24-well plate. 24 h later, the lower surface of the cells was fixed with 4% paraformaldehyde for 20 min and stained with 0.1% crystal violet for 20 min. Finally, five random areas of the lower membrane were photographed with an inverted microscope, and the cells were counted for measurement.

### Wound Healing Assay

Exponentially grown A2780 and SKOV3 cells are inoculated at 85% fusion on a 6-well plate. A pipette (200 uL) is used to make a straight-line scratch in the center of the plate. After gentle rinsing with PBS and changing to a serum-free medium, the width of the scratch was photographed under the microscope at 0 h, 24 h and 48 h at the same location and then measured and analyzed using ImageJ software (v1.8.0).

### Clone Formation Assay

6-well plates were inoculated with 200 cells/mL of A2780 and SKOV3 cells per well. PEI-Pt NCs at 0 ug/mL, 0.05ug/mL and 0.1 ug/mL were added to each cell line, and each well was repeated three times. Colony formation was observed under the microscope every 7 d, and the medium was replenished weekly. Colonies formed after 10 d were fixed with 4% paraformaldehyde for 20 min, then stained with 0.1% crystal violet for 20 min, washed gently with ddH_2_O, dried at room temperature, and then photographed microscopically.

### Tunel Assay

Cells were fixed in 4% paraformaldehyde for 5 min at room temperature and then washed 3 times with phosphate. After washing, the cells were closed with 5% skim milk and 0.1% Triton X-100 for 1h at room temperature, then incubated with tunel primary antibody (1:500; Sigma) for 1h at 4˚C, followed by Alexa Fluor-labeled secondary antibody (1:500; Sigma) was incubated for 1h and washed with PBS. The cells were stained with DAPI at a constant temperature of 4˚C for 10 min. Stained cells were observed under five non-overlapping fields of view (BX60; Olympus Corporation, Tokyo, Japan).

### Statistical Analysis

Statistical analysis was performed using GraphPad Prism 9.0. software. Experimental data were presented as mean ± standard deviation. A T-test was used to compare the mean value between the two groups. One-way ANOVA (One-way ANOVA) was used to compare three and more than three groups. * P<0.05 and **P<0.01 suggest a statistically significant difference.

## Results

### Distribution and Bioimaging of PEI-Pt NCs

The characterization results of PEI-Pt NCs are detailed in **Supplementary Materials** (**Figure S1**). Ovarian cancer A2780 cells and SKOV3 cell line were selected as experimental target cells to examine the bioimaging ability of PEI-Pt NCs (**Figures 2A, B**). Meanwhile, normal ovarian cells IOSE80 were selected as control (**Figure 2C**). Confocal microscopy images of PEI-Pt NCs-like chemotherapeutic drug-labeled A2780 and SKOV3 showed green fluorescent signals (PEI-Pt NCs self-fluorescence) and mainly were distributed around (DAPI blue fluorescence) membrane and partially into the nucleus. Ovarian cancer cell lines tend to endocytose more PEI-Pt NCs than normal cells. After 4 h co-culture, PEI-Pt NCs entered the nucleus of ovarian cancer cells well and expressed higher fluorescence intensity. In addition, we observed the changes in cell morphology and status after adding different drug concentrations of PEI-Pt NCs under a microscope. **Figures 2D–F** shows that PEI-Pt NCs added at 0.05ug/mL and 0.1ug/mL in both normal and cancer cell lines were observed compared to the 0 ug/mL group. It can be observed that normal cells have fewer cell morphological changes compared to ovarian cancer cells. At the same time, it was found that with the increase in drug dose, ovarian cancer cells showed a shrinking and rounding phenomenon, and there was a tendency for apoptotic cells to fragment. Interestingly, our bifunctional chemotherapeutic drug based on PEI-Pt NCs can be used as a fluorescent marker for ovarian cancer cells without the addition of any other fluorophores for bioimaging, and it has specific passive targeting to cancer cells.

### PEI-Pt NCs Inhibit the Proliferation, Migration, and Clone Formation of Ovarian Cancer Cells

It is determined that the PEI-Pt NCs drug can tend to bind to ovarian cancer cells and enter the nucleus, whether it can play an anti-tumor effect in ovarian cancer? First, we treated ovarian cancer cell lines with different concentration gradients of PEI-Pt NCs. **Figure 2G** showed that PEI-Pt NCs are less toxic to normal ovarian cells than ovarian cancer cells. **Figures 2H, I** showed that PEI-Pt NCs had a better inhibitory effect on ovarian cancer cell proliferation compared with the same dose of cisplatin. Subsequently, the results of CCK8 were further verified by the Ki-67 immunofluorescence assay (**Figures 2J, K**). The results showed that the immunofluorescence of Ki-67 after PEI-Pt NCs treatment was significantly reduced, and the proliferation activity of the cells decreased. The results of the two experiments are consistent. Subsequently, we found that PEI-Pt NCs could reduce the migration ability of ovarian cancer cells through cell wound healing assay (**Figure 3A**) and transwell assay (**Figure 3B**) from different angles and different experimental methods (distance and number). In addition, the results of the clone formation assay in **Figure 3C** showed that the monoclonal formation ability of ovarian cancer cells was attenuated by PEI-Pt NCs drug treatment. In conclusion, our bifunctional PEI-Pt NCs drug has apparent antitumor effects on ovarian cancer, including inhibiting its malignant biological behaviors such as proliferation, migration, and clone formation. Therefore, PEI-Pt NCs are expected to be further used as an effective therapeutic drug for ovarian cancer patients through later clinical trials. But whether it affects the apoptosis of ovarian cancer cells also deserves further study.

### PEI-Pt NCs Induces Apoptosis in Ovarian Cancer Cells

We further found that PEI-Pt NCs could promote the apoptosis of ovarian cancer cells by flow cytometry (**Figure 4A**). The apoptosis rate of the drug-treated group was significantly increased. With the increase of drug concentration (0 ug/mL, 0.05 ug/mL, 0.1 ug/mL), the early apoptosis rates in the A2780 cell group were 1.0%, 25.6%, and 28.9%, respectively, and the early apoptosis rates in the SKOV3 cell group were 0%, 2.9%, and 9.4%, respectively. In addition, the conclusion of apoptosis by flow cytometry was further confirmed by the tunel assay (**Figures 4B, C**). The tunel green fluorescence increased with the increase of drug concentration (0 ug/mL, 0.05 ug/mL, 0.1 ug/mL); the three groups were compared with statistical significance (P<0.05). These data suggest that our developed bifunctional PEI-Pt NCs have favorable pro-apoptotic effects in ovarian cancer cell systems and deserve attention. Finally, we used western blotting assay to confirm further that apoptosis-related proteins (P53, Caspase3, BCL-2, and Bax) in cells treated with PEI-Pt NCs were changed with statistical significance. **Figures 4D, E** shows that compared with the untreated group, the protein expressions of P53, Caspase3 and Bax in the PEI-Pt NCs group increased while BCL-2 decreased, and the cell self-results of the two groups remained consistent.

## Discussion

**Figure 1:**
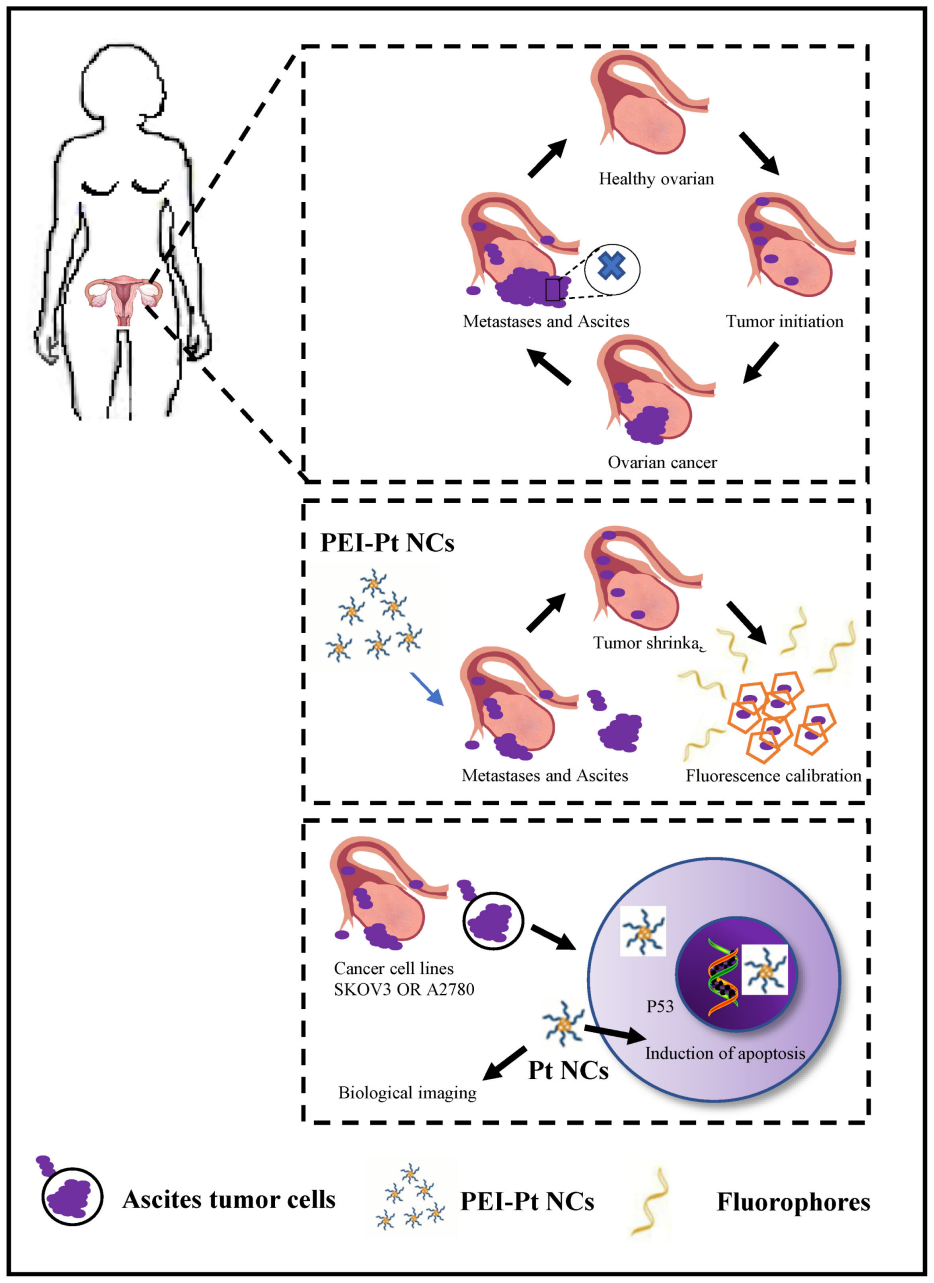
Schematic representation of the process of bioimaging and pro-apoptosis of ovarian cancer cells using fluorescent PEI-coated Pt NCs as fluorescent markers and anti-cancer agents.

**Figure 2:**
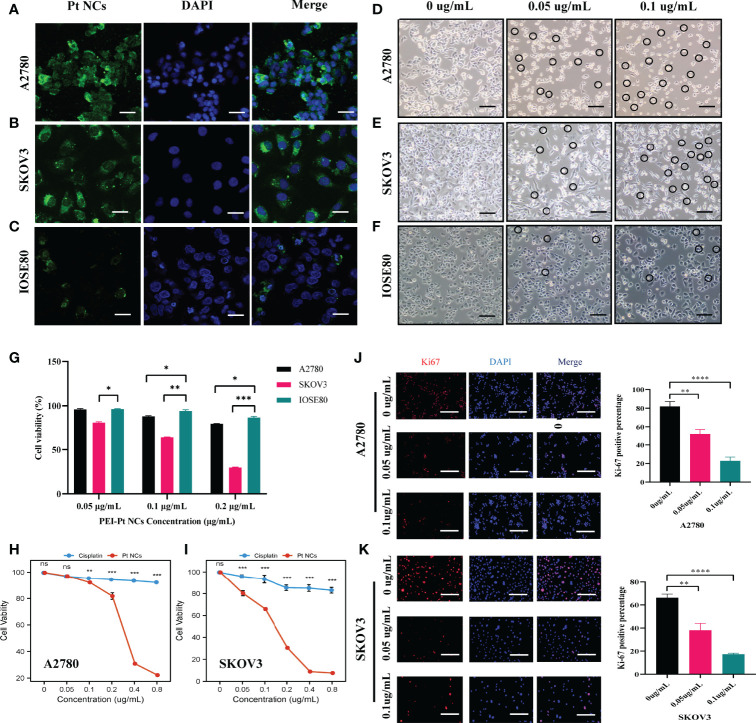
Fields of view taken with a confocal microscope and normal microscope after PEI-Pt NCs treatment. **(A-C)** Confocal microscopy images of A2780SKOV3 and IOSE80 cells after co-culture with PEI-Pt NCs for 4 h. The green fluorescence is the PEI-Pt NCs and the blue fluorescence is the DAPI staining of the nuclei. **(D-F)** Morphological changes of A2780SKOV3 and IOSE80 cells after co-culture with different concentrations of PEI-Pt NCs for 24 h under a general microscope. **(G)** Effects of different concentrations of PEI-Pt NCs on the proliferation of ovarian normal cells and cancer cells. **(H-I)** Effect of treatment with different concentrations of PEI-Pt NCs on the proliferation of ovarian cancer cells. **(H)** Statistical analysis of CCK8 test results for A2780 cell line. **(I)** Statistical analysis of CCK8 test results of SKOV3 cell line. Cells were treated with 0ug/mL, 0.05ug/mL, 0.1ug/mL, 0.2ug/mL, 0.4ug/mL and 0.8ug/mL concentration gradients of cisplatin and PEI-Pt NCs. **(J-K)** Ki67 immunofluorescence staining images and statistical images of A2780 and SKOV3 cell lines treated with different concentrations of PEI-Pt NCs. Ki67 staining is red, DAPI nuclear staining is blue, and Merge is the merged image. Histograms were counted for the number of Ki67 cells. (*p < 0.05, **p < 0.01, ***p < 0.001, ****p < 0.0001.).

**Figure 3:**
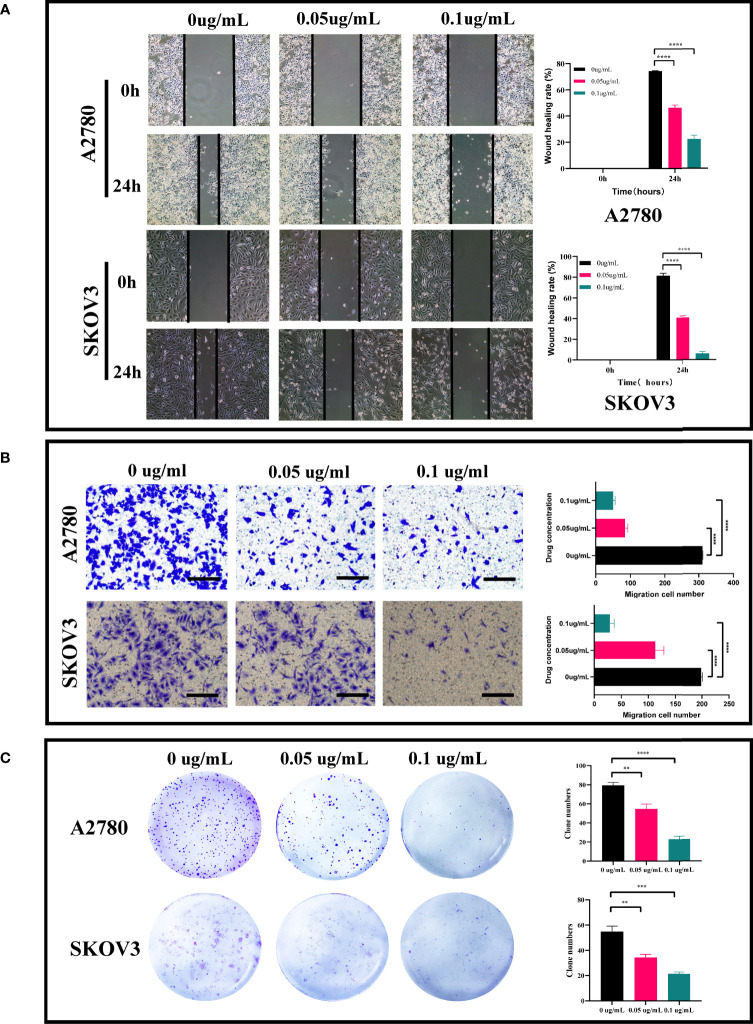
Effect of PEI-Pt NCs treatment on migration and clonogenic ability of ovarian cancer cells. **(A)** Wound healing assay and analysis results of A2780 and SKOV3 cell lines. Cell migration rates were measured at 0h, 24h and 48h, respectively, and histograms show migration rates. **(B)** Transwell assay and analysis results of A2780 and SKOV3 cells. cell migration was measured at 24h after 0.05ug/mL and 0.1ug/mL treatment, respectively, and the histograms show the migration rates. **(C)** Clonogenic assay and analytical results of A2780 and SKOV3 cell lines. The number of clonogenic cell clusters was measured on day 10 after 0.05ug/mL and 0.1ug/mL treatment. (**p < 0.01, ***p < 0.001, ****p < 0.0001).

Applying lipid and polymer nanoparticles to develop platinum-based drugs has opened new ideas for exploring novel medications in recent years. Especially with the rising enthusiasm for exploring nanomaterial-bound small-molecule chemotherapeutics, these novel nanomedicines may reduce side effects and drug resistance and improve the fight against cancer in oncology patients. Based on the nature of the activation of PEI-Pt NCs in various tumor cells, including non-small cell lung cancer and malignant hematologic diseases, it would be interesting to investigate whether this novel bomb is also effective against ovarian cancer (25, 26). We designed a novel platinum anticancer drug, Polyethyleneimine caged platinum nanoclusters (PEI-Pt NCs), a zero-valent platinum based on nanoparticles. Studies have shown that the oxidized state of platinum (corrosive platinum ions) tends to bind to DNA and proteins in the physiological state, leading to DNA damage. **Figure 1** presents a conceptual diagram of the study. Combined with the results of confocal microscopy and morphological changes in drug-treated cells, PEI-Pt NCs may exert antitumor effects by easily binding to the nuclear membrane of ovarian cancer cells and entering the nucleus to release Pt ions that bind to and destroy nuclear DNA (**Figures 2A–G**). Moreover, PEI-Pt NCs has a certain degree of passive targeting of cancer cells; this distinction between normal and cancer cells ensures that PEI-Pt NCs enables selective bioimaging nuclear killing of tumors. This study confirmed that PEI-Pt NCs inhibited ovarian cancer cell proliferation (**Figures 2H–K**), migration (**Figures 3A, B**) and monoclonal formation ability (**Figure 3C**).

**Figure 4:**
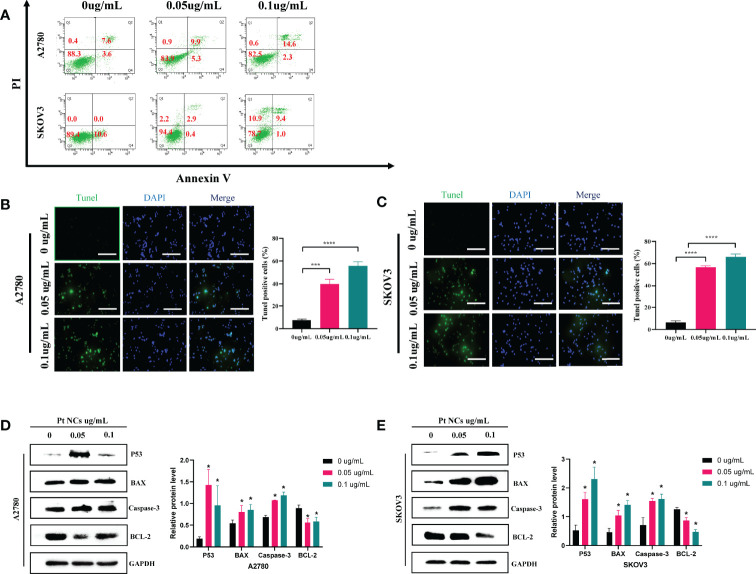
Induction of apoptosis by PEI-Pt NCs in A2780 cells and SKOV3 cells. **(A)** Characteristic fluorescence dot plots showing fluorescence-activated cell sorting (FACS) analysis by membrane-linked protein V staining and PI to determine the cell death status of PEI-Pt NCs after co-incubation at different concentrations (0ug/mL, 0.05ug/mL, 0.1ug/mL) for 4 h. Q2 and Q4 regions represent apoptotic cells. The Q1 region represents cells that have died. The Q3 region represents cells that are alive. **(B)** Tunel apoptosis immunofluorescence staining of cells treated with different concentrations (0ug/mL, 0.05ug/mL, 0.1ug/mL) of PEI-Pt NCs drug in A2780 cell line for 24h. **(C)** Tunel apoptosis immunofluorescence staining of SKOV3 cell line in different concentrations (0ug/mL, 0.05ug/mL, 0.1ug/mL) of PEI-Pt NCs drug-treated cells for 24h. Tunel staining is green, DAPI nuclear staining is blue, and Merge is the merged image. Histograms were calculated for the ratio of apoptotic cells. (*p < 0.05, ***p < 0.001, ****p < 0.0001) **(D)** Histogram of WB results of apoptosis-related genes P53, BAX, Caspase-3 and BCL-2 in A2780 cell line in different concentrations of PEI-Pt NCs drug treatment groups. **(E)** Histogram of WB results of apoptosis-related genes P53, BAX, Caspase-3 and BCL-2 in SKOV3 cell line in different concentrations of PEI-Pt NCs drug treatment groups. * indicates statistical significance.

In addition to this, we also found that PEI-Pt NCs induced apoptosis of ovarian cancer cells (**Figure 4**). It is well known that p53 is an essential component in the apoptotic response and induces transcriptional activation of pro-apoptotic factors such as Caspase-3 and BCL-2-associated X protein (BAX) (27, 28). BCL-2 is a crucial regulator of apoptosis and plays a vital role in apoptosis in ovarian cancer. Our western blotting results preliminarily confirmed that PEI-Pt NCs inhibited the expression of anti-apoptotic protein BCL-2 and the manifestation of pro-apoptotic proteins p53, Caspase-3 and BAX (**Figures 4D, E**). The above experimental results preliminarily found that our bifunctional PEI-Pt NCs had a good effect on inducing apoptosis of ovarian cancer cells. PEI-Pt NCs, highly specialized nanoparticle, provides excellent convenience for ovarian the drug treatment of ovarian cancer. The most exciting thing is its fluorescent properties in addition to its good antitumor effect on ovarian cancer. The innovation and great value of our research are that PEI-Pt NCs may be used as a novel bifunctional drug for calibration. The nanomaterial will release the coated 0-valent platinum to promote the inhibitory pro-apoptotic effects of platinum on ovarian cancer cells. At the same time, fluorescent localization markers can play a specific role in judging tumor cell metastasis and the distribution of metastatic lesions. This new promising drug offers patients new hope and a step toward demystifying ovarian cancer treatment. This is a fascinating study and has significant value in treating ovarian cancer. In conclusion, combining the design of nanomaterials and the research of new small molecule chemotherapeutics makes it no longer a far-fetched vision to develop safe and effective nanomaterials for small molecule chemotherapeutics and conquer ovarian cancer.

## Data Availability Statement

The datasets presented in this study can be found in online repositories. The names of the repository/repositories and accession number(s) can be found in the article/**Supplementary Material**.

## Ethics Statement

The Ethics Committee approved the Harbin Medical University Cancer Hospital (Harbin, China).

## Author Contributions 

XC and XH designed the study. MZ and HY reviewed the raw data and confirmed all raw data’s authenticity. YL and YY performed the analysis. ZS, HL, PC, and FL collected data. MZ drafted the manuscript. XC and XH revised the manuscript. All authors read and approved the final manuscript.

## Funding

This work was supported by grants from the National Natural Science Foundation of China (82073239, 21807121), Henan Province Foundation for University Key Teacher (2021GGJS107), Henan Province Science and Technology Research Project (212102310197) and Harbin Medical University Graduate Practice and Research Innovation Fund (YJSCX2020-51HYD).

## Conflict of Interest

The authors declare that the research was conducted in the absence of any commercial or financial relationships that could be construed as a potential conflict of interest.

## Publisher’s Note

All claims expressed in this article are solely those of the authors and do not necessarily represent those of their affiliated organizations, or those of the publisher, the editors and the reviewers. Any product that may be evaluated in this article, or claim that may be made by its manufacturer, is not guaranteed or endorsed by the publisher.
