# Corrigendum: Biomarking and induction of apoptosis in ovarian cancer using bifunctional polyethyleneimine-caged platinum nanoclusters

**DOI:** 10.3389/fonc.2022.983959

**Published:** 2022-07-20

**Authors:** Mengjun Zhang, Haodi Yue, Yuan Liu, Hao Li, Yue Yin, Zhenxing Sun, Ping Cui, Fei Li, Xiuwei Chen, Xin Huang

**Affiliations:** ^1^ Department of Gynecology Oncology, Harbin, China; ^2^ Department of Center for Clinical Single Cell Biomedicine, Zhengzhou, China; ^3^ Department of Light Chemical Engineering, School of Textiles, Zhengzhou, China

**Keywords:** ovarian cancer, PEI-Pt NCs, apoptosis, bioimaging, nanomedicine

In the published article, there was an error in Figure 3 as published. Figure 3A in the published article showed the results of wound healing assays in ovarian cancer cell lines A2780 and SKOV3 after PEI-Pt NCs co-culture. There was an error in Figure 3A in the published article, the image of wound healing in the ovarian cancer cell line SKOV3 at 0 ug/mL for 24h and 0.05 ug/mL for 0h was wrong. The corrected Figure 3 and its caption: “Figure 3. Effect of PEI-Pt NCs treatment on migration and clonogenic ability of ovarian cancer cells. (A) Wound healing assay and analysis results of A2780 and SKOV3 cell lines. Cell migration rates were measured at 0h and 24h respectively, and histograms show migration rates.“ appear below.

**Figure 3:**
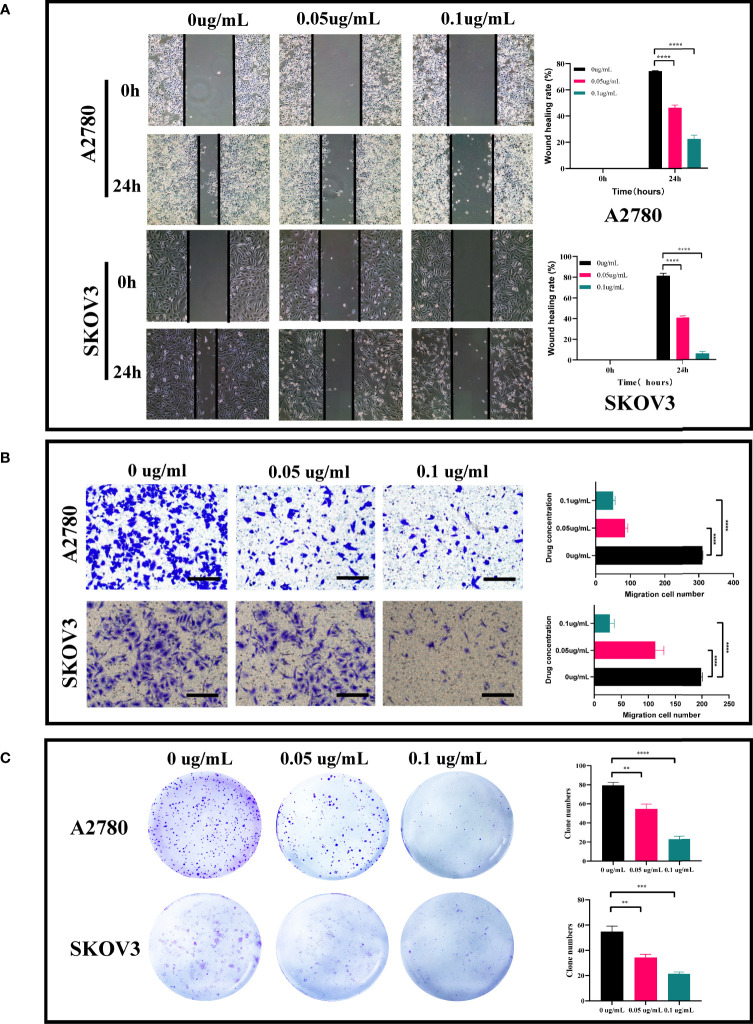
Effect of PEI-Pt NCs treatment on migration and clonogenic ability of ovarian cancer cells. **(A)** Wound healing assay and analysis results of A2780 and SKOV3 cell lines. Cell migration rates were measured at 0h, 24h and 48h, respectively, and histograms show migration rates. **(B)** Transwell assay and analysis results of A2780 and SKOV3 cells. cell migration was measured at 24h after 0.05ug/mL and 0.1ug/mL treatment, respectively, and the histograms show the migration rates. **(C)** Clonogenic assay and analytical results of A2780 and SKOV3 cell lines. The number of clonogenic cell clusters was measured on day 10 after 0.05ug/mL and 0.1ug/mL treatment. (* p < 0.05, ** p < 0.01, **** p < 0.0001).

The authors apologize for this error and state that this does not change the scientific conclusions of the article in any way. The original article has been updated.

## Publisher’s note

All claims expressed in this article are solely those of the authors and do not necessarily represent those of their affiliated organizations, or those of the publisher, the editors and the reviewers. Any product that may be evaluated in this article, or claim that may be made by its manufacturer, is not guaranteed or endorsed by the publisher.

