# Supplementary material for: Biomarking and Induction of Apoptosis in Ovarian Cancer Using Bifunctional Polyethyleneimine-Caged Platinum Nanoclusters
Source: Front Oncol. 2022 Jun 3;12:898917. doi: 10.3389/fonc.2022.898917 (PMC9204061; doi:10.3389/fonc.2022.898917)
Supplement: Supplementary file 1 [file DataSheet_1.doc]

**Supplementary Information**

**Biomarking and induction of apoptosis in ovarian cancer using bifunctional polyethyleneimine-caged platinum nanoclusters**

Mengjun Zhang^1^, Haodi Yue^2^, Yuan Liu^1^, Hao Li^1^, Yue Yin^1^, Zhenxing Sun^1^, Ping Cui^1^, Fei Li^1^, Xin Huang^3^ and Xiuwei Chen^1^

^1^Department of Gynecology, Harbin Medical University Cancer Hospital, Harbin, China.

^2^Department of Center for Clinical Single Cell Biomedicine, Henan Provincial People’s Hospital, Zhengzhou, Henan, China.

^3^Department of Light Chemical Engineering, School of Textiles, Zhongyuan University of Technology, Zhengzhou, Henan, China.

**Corresponding Author 1**: Xiuwei Chen, Ph.D., Department of Gynecology, Harbin Medical University Cancer Hospital, No. 6 Baojian Road, Harbin 150040, China. E-mail: 1427@hrbmu.edu.cn

**Corresponding Author 2**: Xin Huang, Ph.D., Department of Light Chemical Engineering, School of Textiles, Zhongyuan University of Technology, No. 41 Zhongyuan Road (M), Zhengzhou, 450007, China. E-mail: xinhuang@zut.edu.cn

**Contents:**

SI-1: The Synthesis of Polyethylenimine-Caged Platinum Nanoclusters.

SI-2: The Characterization of PEI-caged Pt NCs.

SI-3: References and Notes.

**SI-1:** **The Synthetic Method of** **Polyethylenimine-Caged Platinum Nanoclusters.**

The synthesis of fluorescent polyethylenimine (PEI) caged platinum nanoclusters (Pt NCs) was followed the modified synthetic proposal according to our previous work^1^ as follows: 80 μL Hydrogen hexachloroplatinate (IV) hexahydrate (H_2_PtCl_6_·6H_2_O, 99.9 %) and hyper-branched polyethylenimine (PEI, *Mw* = 10,000, 99.0 %) aqueous solution (400 μL, 15 mM) were added to 3 mL water solution under vigorous stirring to give a final H_2_PtCl_6_ concentration of 0.67 mM. After the complex formed under stirring for over 2 h, the mixture was heated to 95 °C and L-ascorbic acid (L-AA, 99.0 %) was added dropwise. The reaction was allowed to continue 4 days under vigorous stirring. The resultant Pt NCs were centrifuged by ultra-centrifugation (Optima MAX-XP Benchtop Ultracentrifuge, Beckman Coulter, Inc.; × 100,000 g) for 30 min at 4 °C three times and the centrifugal filter unit (cut-off molecule 3,000 and 10,000 g/mol) twice to remove unreacted small molecules.

**SI-2: The** **Characterization of** **PEI-caged Pt NCs.**

Transmission electron microscopy (TEM) was used to determine the size of PEI-caged Pt NCs with JEM-1011 (JEOL, Japan) at room temperature. The average sizes of Pt NCs are 1.6 ± 0.4 nm (Figure S1a) and the Pt NCs exhibited the good dispersity (Figure S1b) which was analyzed by ImageJ 1.80v software. The hydrodynamic size of PEI-caged Pt NCs was tested by a high-performance two angle particle and molecular size analyzer (Zetasizer Nano ZS) using dynamic light scattering (DLS) with ‘NIBS’ optics (Malvern Instruments, United Kingdom). The results suggest the size of PEI-caged Pt NC is 2.01 nm (Figure S1c) which is a little larger than TEM value because DLS determines the hydrodynamic size. The excitation and emission spectra of PEI-caged Pt NCs is evaluated by FP-6200 spectrofluorometer (Shimadzu Corporation, Japan) equipped with a high-power Xenon lamp. The maximum emission wavelength of PEI-caged Pt NCs is 560 nm when the excitation wavelength is 500 nm (Figure S1d). Theses characterization results were consistent with our group's previous work^1^.

**
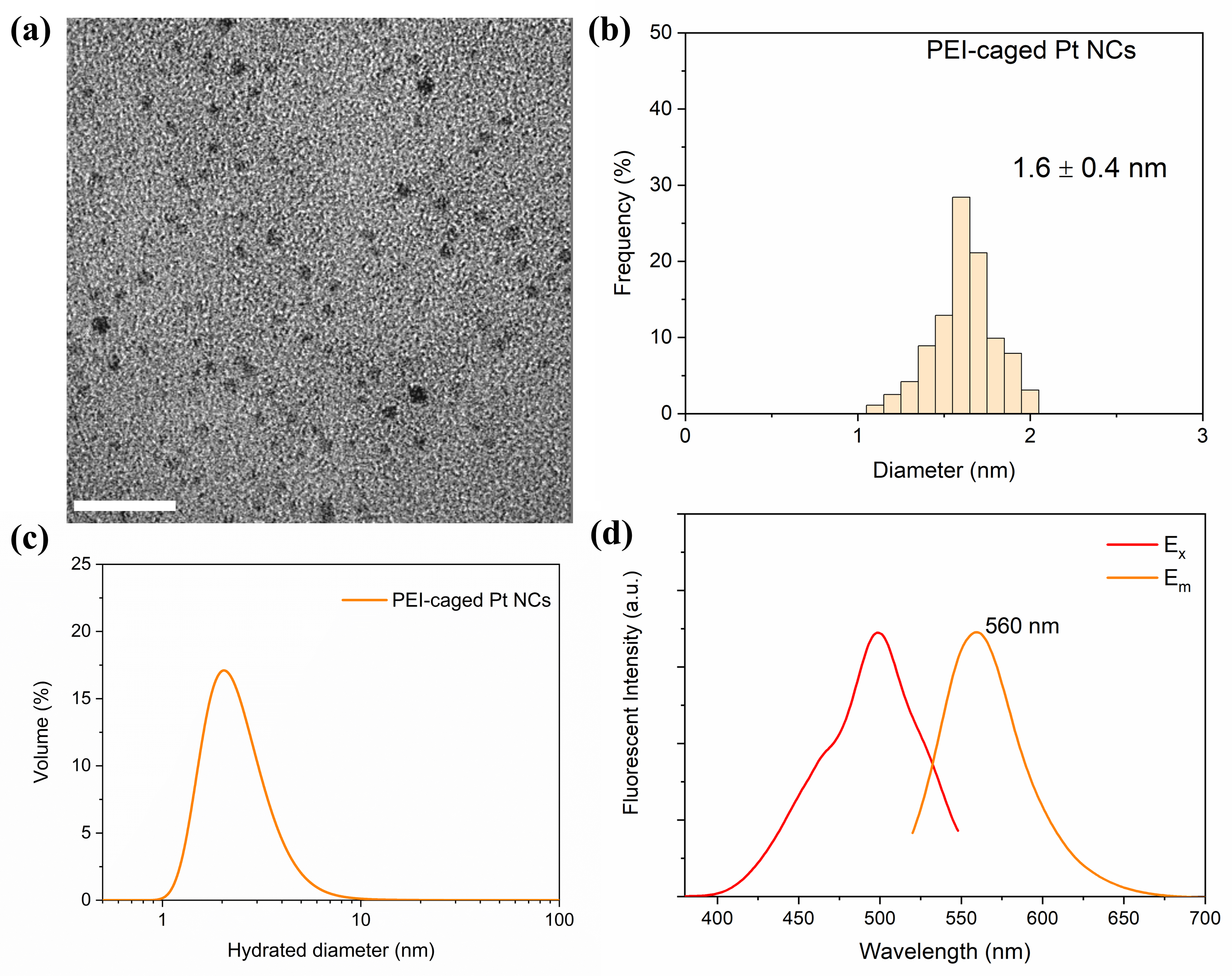
**

Figure S1 (a) TEM micrographs of PEI-caged Pt NCs and (b) corresponding histograms of their size-distribution. Scale bar is 10 nm. (c) DLS result of PEI-caged Pt NCs. (d) Excitation and emission spectra of PEI-caged Pt NCs.

**SI-3: References and Notes.**

1. Chen, X.; Zhou, J.; Yue, X.; Wang, S.; Yu, B.; Luo, Y.; Huang, X., Selective Bio-Labeling and Induced Apoptosis of Hematopoietic Cancer Cells Using Dual-Functional Polyethylenimine-Caged Platinum Nanoclusters. Biochem. Biophys. Res. Comm. 2018, 503 (3), 1465-1470.
